# Validation of a deep learning-based image analysis system to diagnose subclinical endometritis in dairy cows

**DOI:** 10.1371/journal.pone.0263409

**Published:** 2022-01-28

**Authors:** Hafez Sadeghi, Hannah-Sophie Braun, Berner Panti, Geert Opsomer, Osvaldo Bogado Pascottini

**Affiliations:** 1 Department of Reproduction, Obstetrics and Herd Health, Ghent University, Merelbeke, Belgium; 2 Department of Theriogenology, University of Tehran, Tehran, Iran; 3 Oculyze GmbH, Wildau, Germany; 4 Department of Veterinary Sciences, Gamete Research Center, University of Antwerp, Antwerp, Belgium; University of Engineering and Technology Taxila Pakistan, PAKISTAN

## Abstract

The assessment of polymorphonuclear leukocyte (PMN) proportions (%) of endometrial samples is the hallmark for subclinical endometritis (SCE) diagnosis. Yet, a non-biased, automated diagnostic method for assessing PMN% in endometrial cytology slides has not been validated so far. We aimed to validate a computer vision software based on deep machine learning to quantify the PMN% in endometrial cytology slides. Uterine cytobrush samples were collected from 116 postpartum Holstein cows. After sampling, each cytobrush was rolled onto three different slides. One slide was stained using Diff-Quick, while a second was stained using Naphthol (golden standard to stain PMN). One single observer evaluated the slides twice at different days under light microscopy. The last slide was stained with a fluorescent dye, and the PMN% were assessed twice by using a fluorescence microscope connected to a smartphone. Fluorescent images were analyzed via the Oculyze Monitoring Uterine Health (MUH) system, which uses a deep learning-based algorithm to identify PMN. Substantial intra-method repeatabilities (via Spearman correlation) were found for Diff-Quick, Naphthol, and Oculyze MUH (r = 0.67 to 0.76). The intra-method agreements (via Kappa value) at ≥1% PMN (κ = 0.44 to 0.47) were lower than at >5 (κ = 0.69 to 0.78) or >10% (κ = 0.67 to 0.85) PMN cut-offs. The inter-method repeatabilities (via Lin’s correlation) were also substantial, and values between Diff-Quick and Oculyze MUH, Naphthol and Diff-Quick, and Naphthol and Oculyze MUH were 0.68, 0.69, and 0.77, respectively. The agreements among evaluation methods at ≥1% PMN were weak (κ = 0.06 to 0.28), while it increased at >5 (κ = 0.48 to 0.81) or >10% (κ = 0.50 to 0.65) PMN cut-offs. To conclude, deep learning-based algorithms in endometrial cytology are reliable and useful for simplifying and reducing the diagnosis bias of SCE in dairy cows.

## Introduction

Subclinical endometritis (SCE) is the most prevalent uterine disease in dairy cows. It can be defined as the superficial inflammation of the endometrium without signs of purulent discharge in the vagina [1]. Unfortunately, it remains largely undiagnosed by veterinarians and farmers, mainly because of a lack of cow-side diagnostic tests [2]. Considering that SCE significantly impairs reproductive performance, its accurate and fast diagnosis has become essential when striving for successful therapy.

Subclinical endometritis can be diagnosed using histopathology, ultrasonography, and endometrial cytology. Endometrial cytology is an inexpensive and reliable technique, thus considered the most used tool for diagnosing SCE [3, 4]. Endometrial cytology can be performed via cytobrush (CB), low volume uterine lavage, or the cytotape [5–7]. The CB technique is a feasible and robust method that provides high-quality samples and is, as such, the most applied procedure to obtain endometrial cytology samples [7, 8]. After sampling, endometrial cytology slides need to be air-dried and stained. The modified Wright-Giemsa dye is the most used method to stain endometrial cytology slides [7, 9]. However, the Naphthol-AS-D-chloroacetate-esterase (Naphthol) staining is considered as the gold standard to identify polymorphonuclear leucocytes (PMN) since they appear bright red after staining [10, 11]. However, due to its high costs and complex procedure, Naphthol is rarely used in practice.

After staining, endometrial slides are evaluated by manually counting the per cell proportion of PMN under conventional light microscopy. This process is not only laborious and cumbersome, but it is also highly subjective. Even in microscopic evaluations performed by expert screeners, it is difficult to achieve optimal repeatability and reproducibility. Moreover, evaluations are often done several hours (or days) after sampling. Thus, due to its technicality, SCE is rarely diagnosed in the bovine in daily veterinary practice. Therefore, using a non-biased and automated diagnostic system might become advisable to evaluate endometrial cytology slides, tackling the tediousness and subjectivity of the manual counting method.

With the advent of artificial intelligence technologies, deep learning-based algorithms are increasingly applied as a powerful and promising tool for evaluating cytological images [12–15]. One of the main advantages of a deep learning-based system is that no technical knowledge of cell-type recognition is needed. Hence, automation of the PMN counting via deep learning technology may facilitate and stimulate the diagnosis of SCE in daily veterinary practice. Additionally, there is no human bias associated with identifying and counting cells [16]. Furthermore, with their high throughput, such technologies can significantly improve the efficiency of evaluation of PMN by eliminating operator variability and minimizing operation hours. No previous study has examined the use of artificial intelligence for the identification and counting of PMN in endometrial cytology slides. Thus, we aimed to validate the accuracy and efficiency of a state-of-the-art deep learning-based system to assess the per cell proportion of PMN in endometrial cytology slides. To do so, diagnostic values of a deep learning-based automation software were compared with manually counted samples stained with modified Wright-Giemsa and Naphthol staining.

## Materials and methods

### General

This diagnostic test validation study included 116 multiparous Holstein cows from one commercial dairy farm in Flanders, Belgium. Cows were fed a totally mixed ration and milked twice daily in a conventional parlor reaching an average milk production of 10,000 kg/cow/305 days. Endometrial cytology samples were collected in the fifth week postpartum (the procedure is described below). Although the presence of purulent vaginal discharge (PVD) was not used as an exclusion criterion, all animals were clinically healthy (no fever or systemic signs of disease) and not yet inseminated. A veterinarian collected samples during weekly visits. The sampling procedure was approved by the Animal Ethics Committee of the Faculty of Veterinary Medicine at Ghent University (EC 2013/174).

### Sample collection

Endometrial cytology samples were collected using the CB technique (CooperSurgical, Berlin, Germany) as described by Pascottini et al. (2015) [7]. Briefly, a human use Cytobrush Plus GT (CooperSurgical, Berlin, Germany) was adapted to a stainless-steel stylet of a universal insemination gun (Agtech, Manhattan, KS, USA) and covered with a 12ʺ-long Sani-Shield Rod (Agtech, Manhattan, KS, USA). Cows were restrained, and their vulva and perineum were rinsed with fresh water and dried with a paper towel. Next, the CB rod was inserted into the vagina and gently passed through the cervix by rectal handling. Upon reaching the uterine lumen, the CB was exposed and rolled twice against the dorsal part of the uterine body. Then, the CB was retracted into the Sani-Shield Rod and carefully removed from the reproductive tract.

### Preparation and staining of the slides

**Fig 1:**
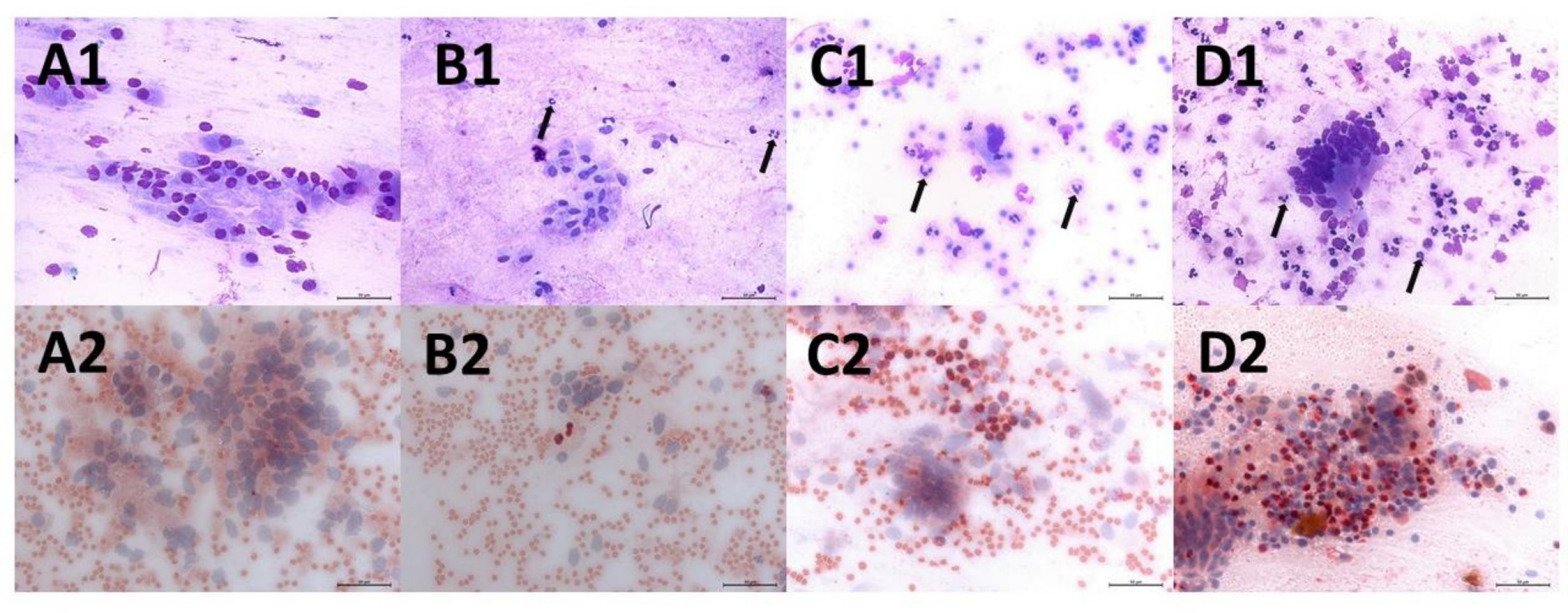
Endometrial cytology smears evaluated by light microscopy (× 400 magnification). Images A1, B1, C1, and D1: Cytology slides stained with the Diff-Quick method, black arrows point polymorphonuclear leucocytes (PMN). Images A2, B2, C2, and D2: Cytology slides stained with the Naphthol method, PMN appearing in bright red. Images A1 and A2 represent 0% PMN; B1 and B2 represent ≥1% PMN; C1 and C2 represent >5% PMN; D1 and D2 represent >10% PMN.

Slides for cytologic examination were prepared by gently rolling the CB onto three different microscope slides (triplicate set of samples) immediately after sampling. All slides were air-dried and placed in a slide box. According to the manufacturer’s instructions, the first set of samples (n = 116) was stained using a commercially available modified Giemsa staining (Diff-Quick, Fisher Diagnostics, Newark, DE, USA) (Fig 1A1–1D1). The second set of samples (n = 116) was stained using Naphthol, which is considered the gold standard to stain PMN since they appear in bright red under the microscope (Fig 1A2–1D2).

Preparation of Naphthol staining was carried out as previously reported by Leder et al. (1970) and Overbeck et al. (2013) [10, 11]. Briefly, two stock solutions were prepared: a substrate solution and a hexasodium solution. For preparing the substrate solution, 3.58 mg of Naphthol-AS-Dichloroacetate (Sigma, ref. no. N0758, St. Louis, USA) was diluted in 0.9 mL of dimethyl sulfoxide (Sigma, ref. no. D5879, St. Louis, USA) and 0.1 mL Triton X-100 (Sigma, ref. no. X100, St. Louis, USA). For the hexasodium solution, a sodium nitrite solution 1 mol/L was prepared by diluting 345 mg of sodium nitrite (Carl-Roth, ref. no. 8604.1, Karlsruhe, Germany) in 5 mL of distilled water. Once the sodium nitrite solution was prepared, pararosaniline hydrochloride (Sigma, ref. no. P3750, St. Louis, USA) was diluted in 3 mL of 1 mol/L HCl (Chem-lab, ref. No. CL05.0311.1000, Zedelgem, Belgium). Then, 0.5 mL of nitrite solution was added to the pararosaniline-HCl solution. The substrate and the hexasodium solution rested for 5 minutes to reach stabilization. To prepare the final Naphthol solution, 1 mL of substrate solution and 0.5 mL of hexasodium solution were added to 100 mL of phosphate-buffered saline (pH = 7.2) and vortexed until a light pink color appeared. Then, slides were incubated for 90 minutes at 37°C in the Naphthol solution. After incubation, slides were rinsed for 2 minutes with tap water and for 5 minutes with distilled water. For counterstaining, slides were submerged in a hemaluin Gill staining solution for 7 minutes. Finally, slides were rinsed with tap water for 10 minutes and distilled water for 5 minutes.

Once the Diff-Quick and the Naphthol slides were dry, coverslips (Marienfeld, Lauda-Königshofen, Germany) were mounted on the stained glass slides using Eukitt (O. Kindler GmbH, Freiburg, Germany) as the mounting medium. Once the mounting medium dried up, Diff-Quick and Naphthol slides were stored in slide boxes until further evaluation.

**Fig 2:**
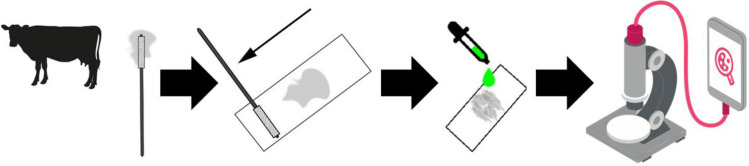
Schematic illustration for the sample preparation process for the Oculyze Monitoring Uterine Health (Oculyze MUH) system. After sampling, the cytobrush was immediately rolled onto a glass slide, air-dried, and placed in a box for transportation. Once at the lab, two drops of fluorescent dye were added, and the slide was subsequently analyzed with a fluorescent microscope connected to a smartphone.

The third set of samples (n = 116) was stained using a ready-to-use fluorescent solution (Oculyze GmbH, Germany), which is a cyanine-based nucleic acid staining emitting green light at 520 nm. To do so, two drops of the fluorescent staining were placed in the center of the microscope glass slide and directly mounted with coverslips (Marienfeld, Lauda-Königshofen, Germany). These slides are referred to as Oculyze Monitoring Uterine Health (Oculyze MUH) slides and were evaluated within 15 min after mounting. A schematic illustration of the sample preparation process for Oculyze MUH is depicted in Fig 2.

### Diff-Quick and Naphthol evaluation

Diff-Quick and Naphthol slides were examined by a single, experienced observer under light microscopy (Kyowa Optical, Tokyo, Japan) at × 400 magnification. The slide examination procedure was repeated twice on two different days (by the same observer) for each slide to assess the intra-method repeatability. The per-cell proportions of PMN were assessed by counting a total of 300 nucleated cells as described by Melcher et al. (2014) [8]. For each examination, the slide number was blinded to the observer before evaluation. The slide number was revealed to the observer only after the assessment of the PMN%, and results were written down in a Microsoft Excel spreadsheet (Microsoft Corp., Redmond, WA). The first examination results were also blinded to the observer when reporting the second examination results (two different Microsoft Excel spreadsheets were used).

### Oculyze MUH system

The Oculyze MUH system is a computer vision algorithm based on deep machine learning. A detailed video depicting the step-by-step guide on how to use Oculyze MUH can be accessed through the following link https://www.youtube.com/watch?v=BIxNHBmc7yc. For the present experiment, a fluorescence microscope (Science ADL-601 F LED, Bresser, Germany) was equipped with a 5MP USB C-Mount Microscope Camera (Banggood.com, China) and connected to a smartphone (Mi A2, Xiaomi, China) with the Oculyze MUH application version 1.2.7 installed (https://play.google.com/store/apps). For image acquisition, the Oculyze MUH application was initialized, and the microscope was adjusted at × 400 magnification. Pictures from the slide were taken in different, randomly selected high-power fields until 300 cells were recognized (automatically counted by the software). The recorded images were uploaded to a cloud-based image analysis platform backend with a specifically deployed computer vision system to analyze the PMN counts of each slide. Within 5 seconds, results were downloaded and displayed in the Oculyze MUH application. Slides were examined twice in a row (in different high-power fields) by the same operator in order to determine the intra-method agreement.

### Bioinformatic approach

**Fig 3:**
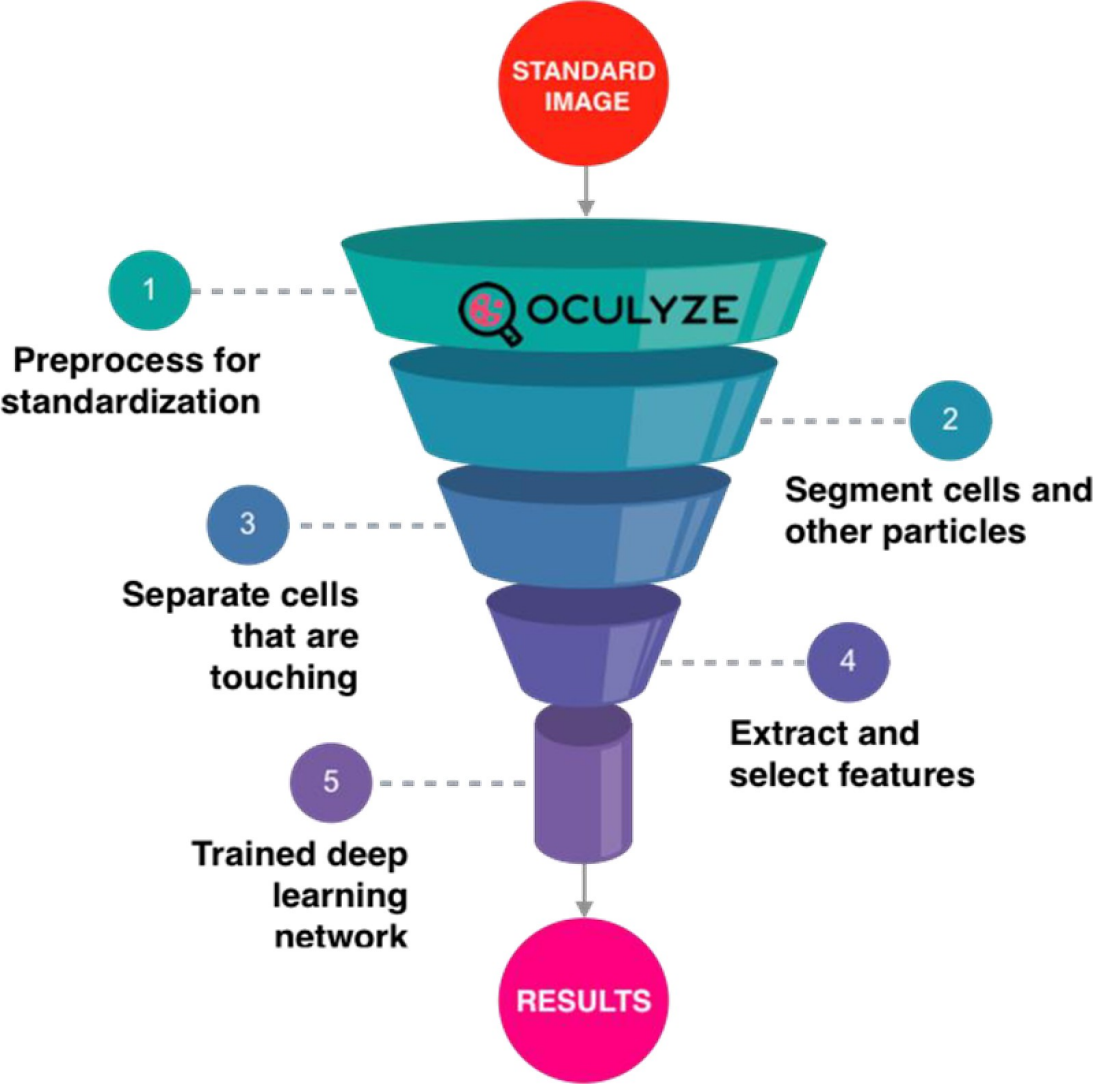
Graphical representation of the levels of image recognition and the analysis process used by the Oculyze Monitoring Uterine Health (Oculyze MUH) system to identify and quantify polymorphonuclear leucocytes (PMN) in endometrial cytology slides.

The captured images were analyzed via the Oculyze MUH software (Oculyze GmbH, Wildau, Germany). The software’s architecture includes three main components: image enhancement preprocessing, deep learning detection analysis, and classification and postprocessing for data formatting and output (Fig 3).

For the image enhancement preprocessing, the red/green/blue (RGB) input of the fluorescent image is first processed. Then, noise reduction is performed by bilateral filtering, and contrast enhancement is applied to isolate cells from the background. The pixel-based processing is carried out using the OpenCV [17] library. To cope with the high range of image noise and distortion variables, a large number of images were previously selected for deep learning training.

The network structure used for the construction of the deep-learning analysis was based on tiny YOLO3, which consists of 13 convolution layers, 6 max-pooling layers, 2 route layers, 1 up-sampling layer, and 2 YOLO layers [18]. Briefly, convolution between images and filters efficiently extracts salient features for object detection. The leaky rectified linear unit (ReLu) was utilized to fire the neurons of the neural network in tiny YOLO3. The max-pooling resizes the feature mapping while increasing the stability and robustness of the network structure. The up-sample layer increases the resolution of the image for scale invariance of the detection. Two route layers concatenated the data from other layers to boost information for the predictions. The YOLO layers performed the final task of defining the bounding boxes and probabilities. The number of epochs was set at 15000. The class probability threshold was set at 0.9. Batch size, image size, and other hyperparameters were set at the tiny YOLO default [18].

**Fig 4:**
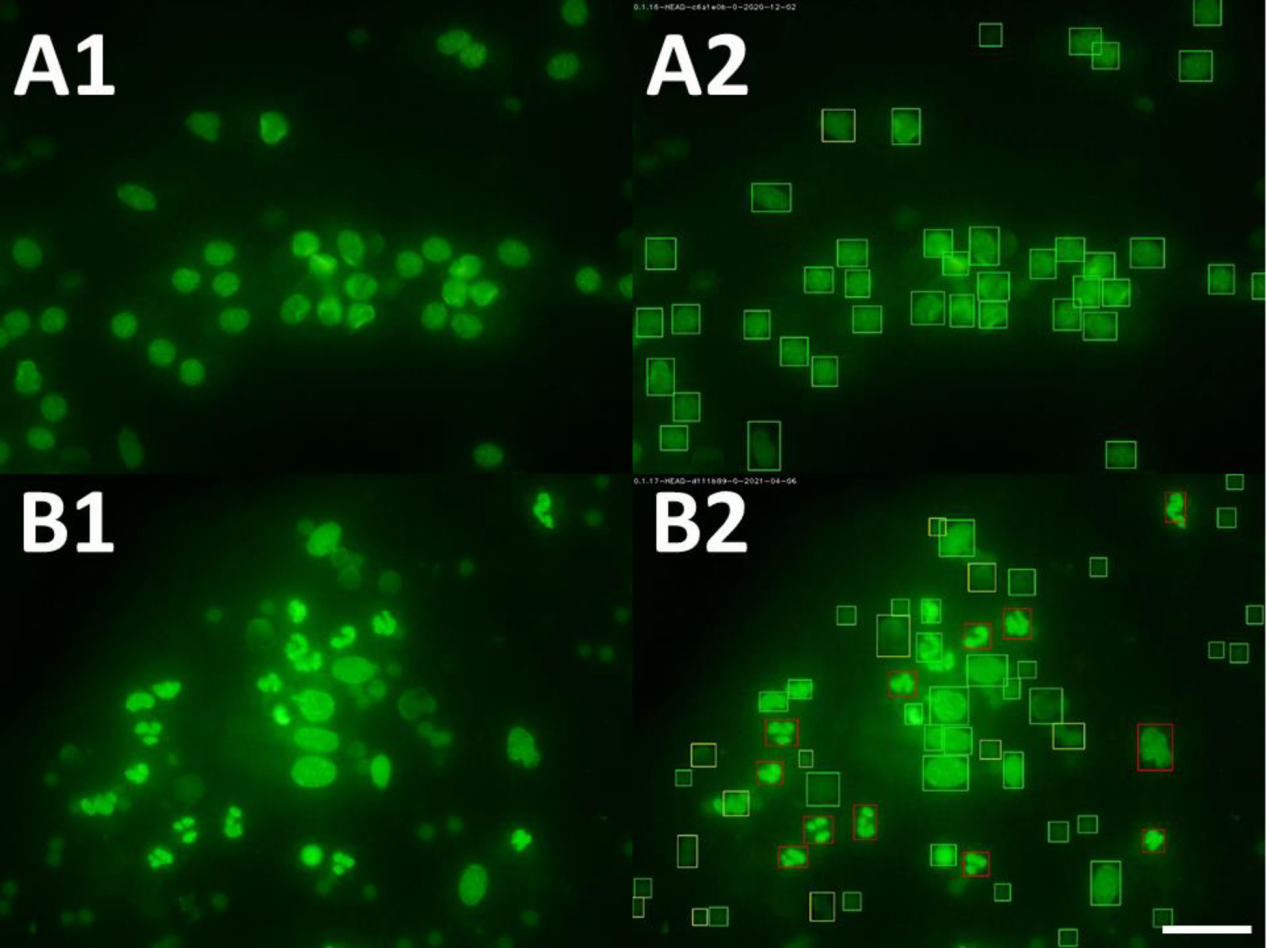
Endometrial cytology smears were evaluated by fluorescence microscopy (× 400 magnification). Cytology slides were stained using a ready-to-use fluorescent solution (Oculyze, GmbH, Germany; A1 and B1). Images were acquired using a smartphone attached to a camera (A2 and B2). Images were analyzed via the Oculyze Monitoring Uterine Health (Oculyze MUH) system, and the detection targets were: polymorphonuclear leukocytes (PMN; red squares), endometrial cells (green squares), and a third-class set to contain disintegrated cells or contaminating particles (yellow squares). Scale bar: 50 μm.

**Fig 5:**
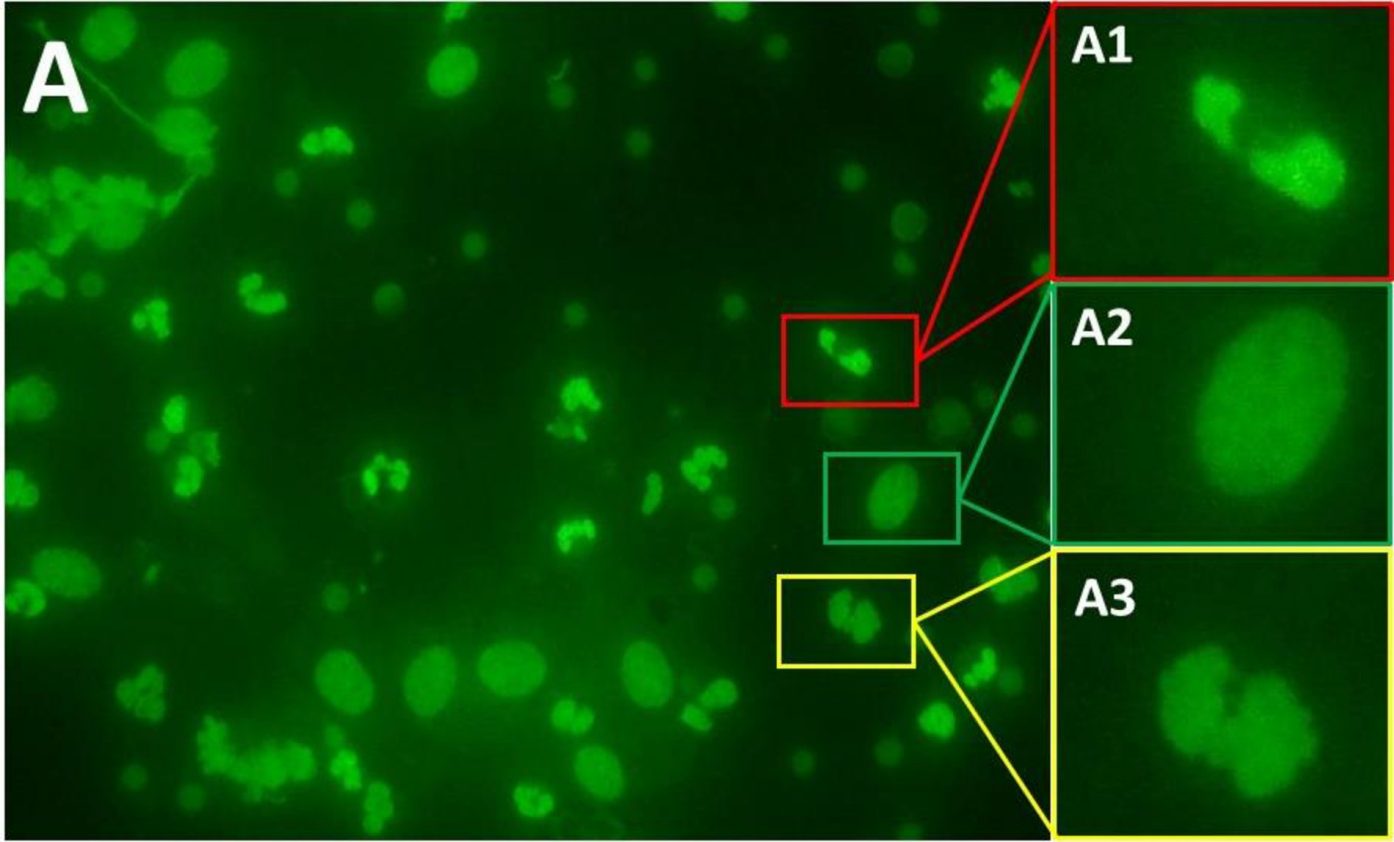
Endometrial cytology smears were evaluated by fluorescence microscopy (× 400 magnification). The cytology slide was stained using a ready-to-use fluorescent solution (Oculyze, GmbH, Germany; A). Image A was acquired using a smartphone attached to a camera. Image A1, A2, and A3 represent different shapes of cell nuclei that were used to train the Oculyze Monitoring Uterine Health (Oculyze MUH) system. Image A1 is a polymorphonuclear leukocyte (red square), image A2 is an endometrial cell (green square), and image A3 is a third-class of shape containing a disintegrated cell or contaminating particle (yellow square).

A Neural Network detection model based on YOLO-V3 [19] was trained with 5000 images containing samples with different challenging attributes (e.g., high blur, low contrast, and clustering cells) for the deep learning analysis. The training images contained a comparable number of cells of each detection target, i.e., given three types of cells, each type ideally representing 33% of the total number of cells found in the images. The detection targets are PMN, endometrial cells, and a third type set of disintegrated cells or contaminating particles (Figs 4 and 5). Given the monochromatic nature of the image and the use of nuclear dye, the main discrimination feature is the shape of the nucleus in relation to the size of the cells. When annotating the dataset for training, the polymorphic nature of the PMN nucleus was annotated as the main target class. Endometrial cells and disintegrated cells are the other classes. The relation to the average cell size in the image is needed to discriminate PMN from clusters of normal endometrial cells. The system scans the image three times using modified versions of the trained YOLO-V3 model to maximize the number of detections. At each step of scanning, local image enhancement is performed to portions of the images affected, for instance, by overexposure or low contrast. Each detection element consists of an array of values represented by a class of detection, confidence score, and location data. The class is defined as an integer for each target defined in the training stage. The confidence value is a score that the Neural Network assigns to an object according to how it matches with a class detection. Finally, the location data is defined as a bounding box containing the cell in x-y coordinates.

The results of the separated scanning steps are joined into a single array of detections, to which non-maximum suppression is applied to discard overlapping results with lower confidence scores. A second outlier filtering is then performed to refine the output confidence value for each detected cell. This consists of a second classification applied to each detected cell through a Neural Network classifier based on Tensorflow [20]. The model for this classification has been obtained by transfer learning based on the Tensorflow Model Inception V3. A large dataset of cells of the different classes has been used to obtain the retrained model. After computer vision analysis of all pictures is finalized, single image results are aggregated, summarizing total cell count and PMN% for all pictures of the respective slide.

### Sample size calculation and statistical analyses

Collected data were merged into a Microsoft Excel file (Microsoft Corp., Redmond, WA). Statistical analyses were performed in R Studio version 4.0.4 (R Inc., Boston, USA). For all evaluation methods described above, PMN counts were reported as a percentage. Moreover, we determined three different PMN cut-off points (≥1, >5, and >10% PMN; represented in Fig 1) as diagnostic thresholds for SCE [5, 21, 22]. Sample size calculations were done using the R packages pwr [23] and kappaSize [24]. One hundred and sixteen samples per experimental group are sufficient to detect a correlation of at least r = 0.3 with a 95% confidence interval (CI) and 80% power. To achieve a correlation of r = 0.61 (sufficient for a validation test), 19 samples per experimental group are necessary. To identify a (substantial) Cohen’s kappa agreement of κ = 0.61 (with a precision of 0.1 on each side) with an expected SCE prevalence of 30%, a total of 93 samples per experimental group is necessary (95% CI and 80% power).

Descriptive statistics were computed using the function summary of the R coding system (package Base). The Spearman correlation test on the percentage of PMN was calculated within each evaluation method to assess the intra-method repeatability (function cor of the package Hmisc) [25]. Lin’s concordance correlation coefficient (CCC, ρc) was calculated using the function epi.ccc of the package epi.R [26] to assess the inter-method agreement on the percentage of PMN among evaluation methods. The function confusionMatrix of the package caret [27] was used to determine Cohen’s kappa agreement, sensitivity (Se), specificity (Sp), positive predictive value (PPV), negative predictive value (NPV), and accuracy to assess the agreement within and among evaluation methods at three different PMN cut-off points (≥1, >5, and >10% PMN). The CCC was interpreted as: 1 reflects total positive correlation, 0 no correlation, and −1 total negative correlation [28]. The κ value agreement was interpreted as: less than 0.21 poor agreement, 0.21 to 0.40 fair agreement, 0.41 to 0.60 moderate agreement, 0.61 to 0.80 substantial agreement, and greater than 0.80 almost perfect agreement [29]. For the intra-method agreements, the first read was considered as the golden standard. For the inter-method agreements, the golden standard was Naphthol, or in its absence, it was Diff-Quick. Correlation and Bland-Altman plots were created to visualize the comparison within and among evaluation methods (packages ggplot2 and epi.R) [26, 30].

## Results

### Descriptive statistics

From the total set of 348 cytology slides, fourteen were excluded due to a low-quality or an insufficient number of cells (< 300 cells). From the remaining 334 slides, 116 Naphthol, 110 Diff-Quick, and 108 were evaluated by the Oculyze MUH. Descriptive data of PMN proportions are shown in Table 1. All staining methods yielded similar PMN proportions resulting in a comparable endometritis prevalence among the 3 different groups.

**Table 1:** Descriptive statistics and subclinical endometritis incidences at different polymorphonuclear leukocytes (PMN) thresholds from endometrial cytology samples collected using the cytobrush technique in the fifth week postpartum from Holstein cows and evaluated by three different methods.

Method	Total samples	Mean	SD	Range	≥ 1% PMN threshold	˃ 5% PMN threshold	>10% PMN threshold
Naphthol	116	4.89	9.73	0–50	61.2% (n = 71)	29.3% (n = 34)	12% (n = 14)
Diff-Quick	110	6.02	15.77	0–90	56.3% (n = 62)	17.2% (n = 19)	8.1% (n = 9)
Oculyze MUH	108	5.17	8.88	0–50	67.5% (n = 73)	25% (n = 27)	8.3% (n = 9)

Values expressed as percentage of PMN. SD is standard deviation.

### Correlations and agreements within evaluation methods

**Fig 6:**
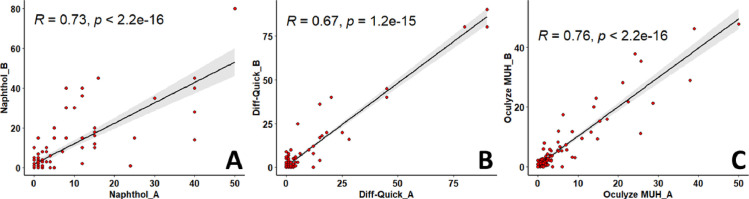
Correlation plots illustrating the intra-method repeatability of polymorphonuclear leukocytes (PMN) percentages of endometrial cytology samples. Naphthol (A) and Diff-Quick (B) slides were evaluated twice by one single observer on different days under light microscopy (× 400 magnification). Oculyze Monitoring Uterine Health system (Oculyze MUH) slides (C) were assessed twice by using a fluorescence microscope connected to a smartphone and analyzed with a deep learning-based algorithm to identify PMN. The black diagonal lines and the gray shadings represent the regressions and the 95% confidence intervals between each read.

The correlation coefficients of PMN% within evaluation methods were all r > 0.6 and *P* < 0.01 (Fig 6). Interestingly, the correlation coefficient of the PMN% within the Oculyze MUH system (r = 0.76) was slightly higher than for Naphthol (r = 0.73) and Diff-Quick (r = 0.67). The intra-method agreements, Se, Sp, PPV, NPV, and accuracies using ≥1, >5, and >10% PMN cut-off points are shown in Table 2. For all evaluation methods, the intra-method agreements at ≥1% PMN (κ = 0.44 to 0.47) were lower than when using >5 (κ = 0.69 to 0.78) or >10% (κ = 0.67 to 0.85) PMN cut-off points (Table 2).

**Table 2:** Intra-method diagnostic characteristics and Cohen’s Kappa values for agreement beyond chance of binomial outcomes for the diagnosis of subclinical endometritis using distinct endometrial cytology evaluation methods and polymorphonuclear leukocytes (PMN) thresholds. Samples were collected using the cytobrush technique in the fifth week postpartum from Holstein cows.

Method^1^	PMN threshold	Sensitivity	Specificity	PPV^2^	NPV^2^	Accuracy	Kappa
Naphthol	≥ 1%	0.71	0.74	0.64	0.80	0.73	0.44
Diff-Quick		0.67	0.77	0.72	0.72	0.72	0.45
Oculyze MUH		0.63	0.83	0.65	0.82	0.76	0.47
Naphthol	> 5%	0.97	0.66	0.87	0.91	0.88	0.69
Diff-Quick		0.97	0.70	0.92	0.89	0.91	0.74
Oculyze MUH		0.96	0.80	0.92	0.88	0.91	0.78
Naphthol	˃ 10%	0.95	0.68	0.92	0.78	0.90	0.77
Diff-Quick		0.96	0.93	0.98	0.82	0.96	0.85
Oculyze MUH		0.98	0.82	0.96	0.93	0.96	0.85

^1^Samples were evaluated twice at different days by a single observer. The first read was considered as the golden standard.

^2^Positive predictive value (PPV) and negative predictive value (NPV).

### Agreements among evaluation methods

**Fig 7:**
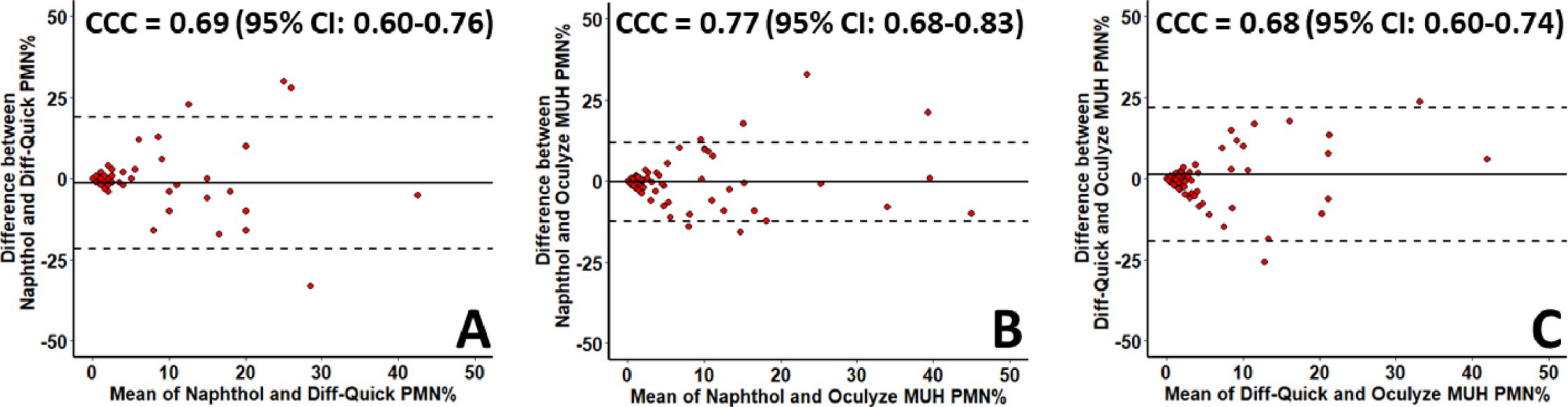
Bland-Altman plots illustrating the relationship of polymorphonuclear leukocytes (PMN) percentages (%) of endometrial cytology slides evaluated via Naphthol, Diff-Quick, and Oculyze Monitoring Uterine Health (Oculyze MUH) system. Lin’s concordance correlation coefficients (CCC) with their respective 95% confidence interval (CI) on the percentage of PMN between evaluation methods are shown on the top of each plot. The solid horizontal lines represent the mean difference, and the dashed lines represent the upper and lower limits of difference.

Graphical representations of the relationships among evaluation methods are shown in the Bland-Altman plots of Fig 7. The CCC of PMN% between Naphthol and Oculyze MUH was *ρ*_c_ = 0.77 (95% CI: 0.68–0.83), which was slightly higher than between Diff-Quick and Oculyze MUH (*ρ*_c_ = 0.68, 95% CI: 0.60–0.74) or between Naphthol and Diff-Quick (*ρ*_c_ = 0.69, 95% CI: 0.60–0.76). In general, the agreements among evaluation methods when using ≥1% PMN as SCE cut-off were low (κ = 0.06 to 0.28), while the agreements for all evaluation methods increased at cut-offs >5 (κ = 0.48 to 0.81) and >10% PMN (κ = 0.50 to 0.65) (Table 3).

**Table 3:** Inter-method diagnostic characteristics and Cohen’s Kappa values for agreement beyond chance of binomial outcomes for the diagnosis of subclinical endometritis using distinct endometrial cytology evaluation methods and polymorphonuclear leukocytes (PMN) thresholds. Samples were collected using the cytobrush technique in the fifth week postpartum from Holstein cows.

Referent method^1^	Test method^1^	PMN threshold	Sensitivity	Specificity	PPV^2^	NPV^2^	Accuracy	Kappa
Naphthol	Diff-Quick	≥ 1%	0.61	0.66	0.59	0.68	0.64	0.28
Naphthol	Oculyze MUH		0.42	0.77	0.60	0.62	0.61	0.20
Diff-Quick	Oculyze MUH		0.48	0.58	0.36	0.70	0.55	0.06
Naphthol	Diff-Quick	> 5%	0.95	0.89	0.97	0.80	0.94	0.81
Naphthol	Oculyze MUH		0.94	0.69	0.90	0.81	0.88	0.67
Diff-Quick	Oculyze MUH		0.92	0.52	0.86	0.66	0.83	0.48
Naphthol	Diff-Quick	˃ 10%	0.94	0.70	0.94	0.70	0.90	0.65
Naphthol	Oculyze MUH		0.91	0.64	0.94	0.52	0.87	0.50
Diff-Quick	Oculyze MUH		0.92	0.69	0.95	0.56	0.89	0.55

^1^Samples were evaluated at different days by one single observer.

^2^Positive predictive value (PPV) and negative predictive value (NPV).

## Discussion

Deep learning-based technologies have been applied to use automated image analysis of cytology samples in human medicine [14, 15, 31, 32]. For the first time in reproductive veterinary medicine, we showed that a deep learning-based software combined with a fluorescent microscope connected to a smartphone could accurately and efficiently identify and quantify PMN in endometrial cytology specimens harvested in cattle. The Oculyze MUH system presented a slightly higher intra-method repeatability than Naphthol or Diff-Quick. Moreover, the inter-method agreement between Naphthol (considered the gold standard to stain PMN) and Oculyze MUH was slightly higher than Naphthol and Diff-Quick. The inter- and intra-agreements were similar among evaluation methods, and when using ≥1% PMN, the diagnostic agreements were all weak. The Oculyze MUH system is at least as efficient as Naphthol and Diff-Quick but has the advantage of simplifying and minimizing a potential bias to diagnose SCE in dairy cows.

Substantial intra-method repeatabilities were obtained for all methods evaluated in the present study. Although differences were minimal, the Oculyze MUH system had a slightly higher intra-method repeatability than Naphthol and Diff-Quick. Other studies [5, 33, 34] found intra-observer correlations ranging from 0.77 to 0.85, similar to the values obtained here. Nevertheless, the intra-method agreements at ≥1% PMN cut-off were only moderate for all tested methods. However, when increasing the cut-off point to >5% PMN, we obtained substantial agreements, similar to those reported by Dubuc et al. (2010). Interestingly, agreement shifted to ‘almost perfect’ when the cut-off point was set at >10% PMN. Thus, our results suggest that the Oculyze MUH system is repeatable and accurate to automatically count PMNs with a substantial accuracy to diagnose SCE starting at the 5% PMN threshold.

To assess the Oculyze MUH system’s efficiency, its PMN% outcome was compared to both the golden standard method to stain PMN (Naphthol) and the most used method to stain cytology slides (Diff-Quick). The agreements (ρc) between the Oculyze MUH system and the other two methods were substantial. This confirms that the deep learning approach used by Oculyze MUH was able to reach the level of an expert screener to identify and count PMN accurately. However, the κ for the inter-method agreement at different PMN% cut-offs were variable. Like the intra-method repeatability, better agreements (κ) among evaluation methods were achieved when using a higher threshold of PMN. Compared to Naphthol as the golden standard, the Oculyze MUH system at cut-off point ≥1% PMN resulted in low Se, PPV, and NPV. This means that the deep learning-based system yields a relatively large proportion of false-positive readings. This may lead to overestimating the true prevalence of SCE by the Oculyze MUH system, as shown in Table 1. Nevertheless, it is essential to mention that the agreement (κ) at ≥1% PMN between Naphthol and Diff-Quick was also weak. In contrast, when using the >5 or 10% PMN cut-off points, the agreements between Naphthol and Oculyze MUH improved, and their Se and PPV were high. These findings are consistent with the study of Dimauro et al. (2019), in which a deep learning system was used to identify neutrophils and eosinophils in human nasal cytology samples with a Se of 0.97 and 1, respectively [31].

Apart from the novelty of the deep learning-based system, it should be noted that the Oculyze MUH is still under development. Therefore, the system might incorrectly classify broken cells and background (debris or contaminating particles) as PMN. This may be the reason for the weak results obtained in the present study while using the 1% PMN cut-off. However, the benefit of a deep learning approach itself is its continuous process of improvement which can only be reached by analyzing an extensive set of images from different qualities [15, 35]. Therefore, it is essential to continuously train the system to the greatest extent possible. It can be expected that the more data is being analyzed, the more accurate the results will become. Albeit reading endometrial cytology slides via the Oculyze MUH reduces human bias, it is worth mentioning that selecting the area of interest to acquire the images to be analyzed is not entirely free of bias. The Oculyze MUH system can eventually be further improved by using a whole slide image algorithm to eliminate the operator selection bias.

## Conclusions

The Oculyze MUH system presented substantial intra-method repeatability. Nevertheless, the intra- and inter-method agreements at ≥1% PMN cut-off to diagnose SCE were weak to moderate for all tested methods. Adequate agreements between Naphthol and Oculyze MUH were obtained at >5 or >10% PMN cut-off points, which are generally applied as reliable diagnostic thresholds to diagnose SCE in dairy cows. Results of the present study are encouraging, and the application of the deep learning technology in endometrial cytology is a promising tool to simplify and reduce bias when diagnosing SCE, with results at least as good as Naphthol or Diff-Quick. However, to improve the performance and robustness of the Oculyze MUH, an expansion of the dataset to train the system with an extensive set of images is warranted.

## Supporting information

S1 Data (CSV)
